# Cell fate determination in cisplatin resistance and chemosensitization

**DOI:** 10.18632/oncotarget.8110

**Published:** 2016-03-16

**Authors:** Khanh V. Luong, Ling Wang, Brett J. Roberts, James K. Wahl, Aimin Peng

**Affiliations:** ^1^ Department of Oral Biology, College of Dentistry, University of Nebraska Medical Center, Lincoln, NE 68583, USA

**Keywords:** chemoresistance, cell fate, cisplatin, Mg132, caffeine

## Abstract

Understanding the determination of cell fate choices after cancer treatment will shed new light on cancer resistance. In this study, we quantitatively analyzed the individual cell fate choice in resistant UM-SCC-38 head and neck cancer cells exposed to cisplatin. Our study revealed a highly heterogeneous pattern of cell fate choices in UM-SCC-38 cells, in comparison to that of the control, non-tumorigenic keratinocyte HaCaT cells. In both UM-SCC-38 and HaCaT cell lines, the majority of cell death occurred during the immediate interphase without mitotic entry, whereas significant portions of UM-SCC-38 cells survived the treatment via either checkpoint arrest or checkpoint slippage. Interestingly, checkpoint slippage occurred predominantly in cells treated in late S and G2 phases, and cells in M-phase were hypersensitive to cisplatin. Moreover, although the cisplatin-resistant progression of mitosis exhibited no delay in general, prolonged mitosis was correlated with the induction of cell death in mitosis. The finding thus suggested a combinatorial treatment using cisplatin and an agent that blocks mitotic exit. Consistently, we showed a strong synergy between cisplatin and the proteasome inhibitor Mg132. Finally, targeting the DNA damage checkpoint using inhibitors of ATR, but not ATM, effectively sensitized UM-SCC-38 to cisplatin treatment. Surprisingly, checkpoint targeting eliminated both checkpoint arrest and checkpoint slippage, and augmented the induction of cell death in interphase without mitotic entry. Taken together, our study, by profiling cell fate determination after cisplatin treatment, reveals new insights into chemoresistance and suggests combinatorial strategies that potentially overcome cancer resistance.

## INTRODUCTION

Genotoxic agents are often utilized in cancer therapy because these drugs cause DNA damage, which, in turn, induce apoptosis and other cell death pathways [1, 2]. Cancer cells can be particularly vulnerable to DNA damage as they actively undergo DNA replication and cell division. However, the therapeutic benefit of chemotherapy is limited in many clinical cases due to intrinsic or acquired resistance of tumor cells to DNA damage. Thus, it has been suggested that targeting the cellular DNA damage response (DDR) may offer a valuable tool to improve the therapeutic window and effectiveness of chemotherapy [3, 4].

Among the most successful and commonly used chemotherapeutic drugs are cisplatin (cis-diamminedichloroplatinum) and other platinum-based drugs. Over the past decades, cisplatin and its variants have been prescribed for an estimated 10 to 20 percent of all cancer patients. The use of cisplatin in the treatment of testicular cancer improved the cure rate from 10% to 80%. Cisplatin is also broadly used for a wide range of other solid tumors, including those of lung, breast, ovarian, head and neck, etc. However, the efficacy of cisplatin in these other solid tumors appears less satisfactory, as many tumors either exhibit resistance to cisplatin or relapse despite initial response [5, 6].

Like other genotoxic drugs or radiation, cisplatin exerts cytotoxicity by inducing DNA damage. Specifically, cisplatin binds DNA and causes DNA inter- or intra-strand crosslinking, a form of DNA damage that blocks DNA replication and transcription [5, 6]. The occurrence of DNA damage quickly activates the DDR, a conserved mechanism evolved in eukaryotic cells to govern genomic integrity. The DDR encompasses various lesion-specific DNA repair pathways, and a sophisticated signaling network that activates the cell cycle checkpoint and cell death [2, 7]. At the center of the DDR pathway are the phosphoinositide 3-kinase-related kinases (PIKK) ATM and ATR. Activation of ATM and ATR by DNA damage results in phosphorylation of dozens of physiologic substrates that control various pathways including DNA repair, checkpoint control, and apoptosis [8]. For example, ATM and ATR activate the checkpoint kinases Chk1 and Chk2, which phosphorylate and inactivate Cdc25, an activator of cyclin-dependent kinases (Cdks), and thereby prevent Cdk activation and cell cycle progression [9].

The ultimate result of DDR activation can be either cell survival or cell death, and the choice between them may essentially dictate the outcome of cancer therapy. In fact, several distinct cell fate choices should be considered. First, cell death can be induced, as the desired outcome that leads to therapeutic benefit. Alternatively, the cell may cease proliferation via sustained activation of the DNA damage checkpoint. Although this cell fate choice halts the growth of tumor cells, these cells may re-enter cell cycle progression after acquiring additional changes. Finally, and perhaps of the worst possibility, cancer cells may continue cell proliferation despite treatment.

In this study we use automated time-lapse microscopy to quantitate the profile of cell fate determination in resistant cancer cells treated with cisplatin. Our study revealed a heterogeneous and complex pattern of cell fate determination in these cancer cells. These results suggested the potential cause of cell protection via both checkpoint activation and checkpoint slippage. Interestingly, our analyses also revealed new insights into how targeting mitotic exit and the DNA damage checkpoint can alter the pattern of cell fate choices to enhance treatment efficacy.

## RESULTS

### Diverse cell fate choices in chemoresistant cancer cells

**Figure 1:**
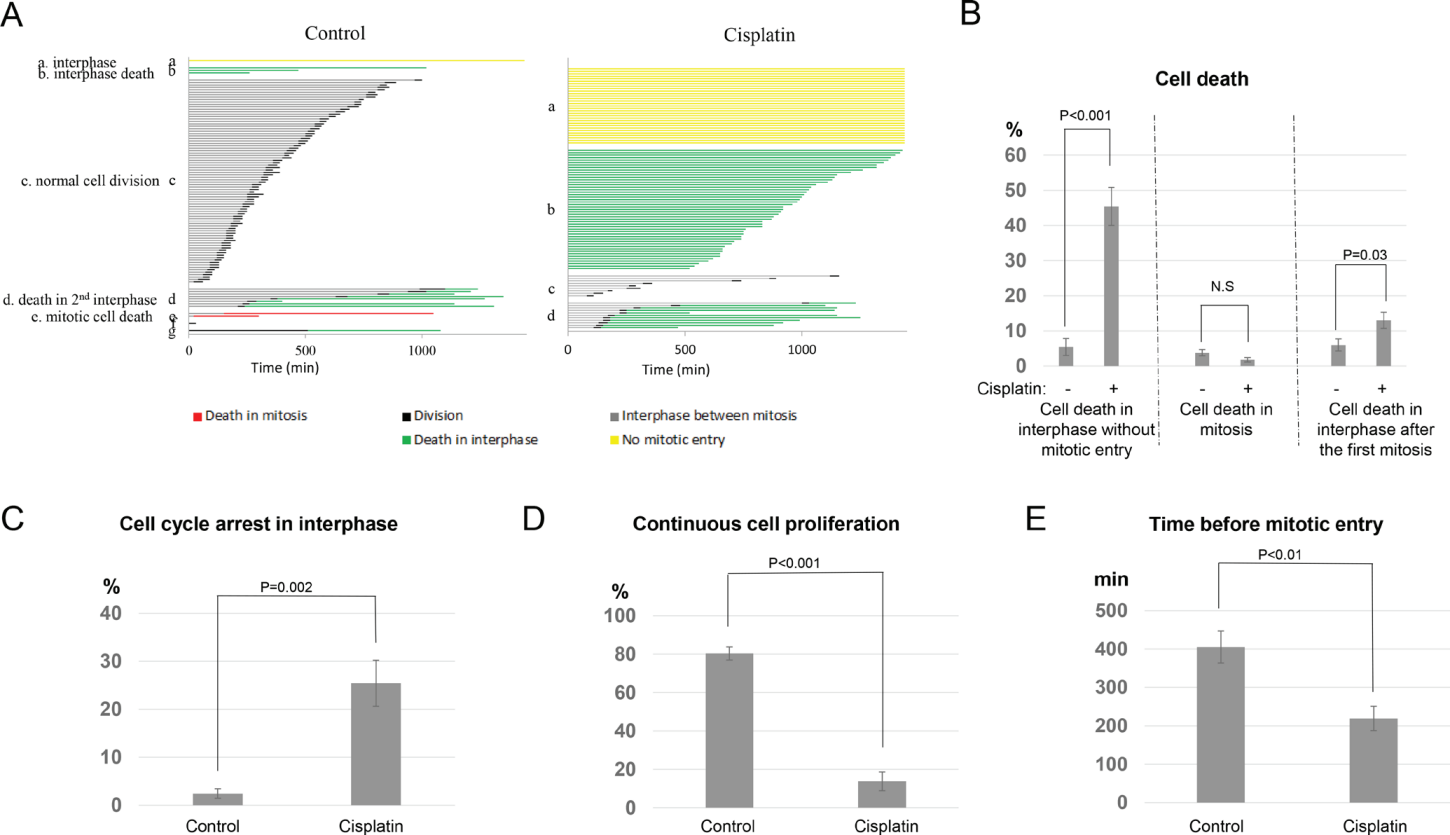
Diverse cell fate choices in resistant cancer cells treated with cisplatin (**A**) As described in Materials and Methods, cell fate profiles of UM-SCC-38 cells treated with or without cisplatin were quantified. A representative experiment is shown. Each horizontal line represents one cell, with the length of the line corresponding to the duration of a given behavior. The color of the line represents a specific cell behavior as indicated. The y-axis is organized to reflect various cell fates: a. interphase (without mitotic entry); b. interphase cell death; c. normal cell division; d. cell death in the 2nd interphase; e. mitotic cell death. (**B**) The induction of cell death by cisplatin in UM-SCC-38 cells. The percentages of cells underwent interphase cell death without mitotic entry, death in mitosis, or death in the subsequent interphase following the first mitosis are shown. UM-SCC-38 cells without cisplatin treatment were included as a control. In all panels, the mean values and standard errors were calculated from multiple independent experiments, as described in Materials and Methods. *P*-value > 0.05 is considered non-significant (N.S). (**C**) UM-SCC-38 cells were treated with or without cisplatin as indicated. The percentages of cells that were arrested in interphase are shown. (**D**) UM-SCC-38 cells were treated with or without cisplatin as indicated. The percentages of cells that exhibited continued cell proliferation are shown. (**E**) The length of interphase (in minutes) prior to mitotic entry is shown in the control and cisplatin-treated UM-SCC-38 cells.

To shed new light on cisplatin resistance, live cell imaging was performed to determine the initial fate of UM-SCC-38 cells after cisplatin exposure (Figure S1 and S2). UM-SCC-38 cell line was selected because this head and neck squamous cell carcinoma (HNSCC) has been previously characterized to be resistant to cisplatin treatment [10, 11]. The majority of unperturbed UM-SCC-38 cells underwent normal cell division, while a dramatically different cell fate profile existed in the presence of cisplatin (Figure 1A). As expected, a significant induction of cell death was observed in cells exposed to cisplatin. Cell death was further investigated for the cell cycle stage in which it occurred (Figure 1B). For example, death in interphase defined those cells that died in the immediate interphase without mitotic entry; death in mitosis characterized those that entered mitosis and died during mitosis; and finally, some cells died in the second interphase after mitotic entry and exit. Interestingly, the majority of cell death (45% of all cells) induced by cisplatin occurred in interphase without mitotic entry (Figure 1B). A moderate increase (to 13%) was documented in cell death in interphase after the first mitosis, but no increase was seen in the portion of mitotic cell death (Figure 1B). Therefore, although mitotic cell death has been implicated in chemotherapy, e.g. via mitotic catastrophe, it did not appear to play a significant role in the treatment of UM-SCC-38 cells with cisplatin.

Consistent with the previously characterized chemoresistance of UM-SCC-38 cells, significant portions of cells survived the treatment. As shown in Figure 1C, approximately 25% of cells remained in the interphase throughout the 24-hour period, compared to 2% in the control group. Presumably, this portion of cells were arrested in interphase due to the activation of the DNA damage checkpoint. The activation of DNA damage checkpoint after cisplatin treatment was consistent with previous studies [5, 12–14], and confirmed by the induction of Chk1 and Chk2 phosphorylation (Figure 5A). Moreover, an average 14% of cells underwent continuous cell cycle progression despite cisplatin treatment (Figure 1D). Thus, this portion of cells escaped the induction of cell death and checkpoint arrest. This cell fate choice is classified as “checkpoint slippage”, as implicated in previous studies [15–18]. The nature of checkpoint slippage is not fully understood. In principle, the deficiency of checkpoint activation can lead to continued cell division after DNA damage. Alternatively, the checkpoint may be initially activated but de-activated subsequently due to DNA repair, or hyperactivation of checkpoint recovery or adaptation mechanisms [17–19].

Interestingly, cells in the group of checkpoint slippage entered mitosis in approximately 3.5 hours after cisplatin-treatment (Figure 1E). By comparison, mitotic entry in unperturbed cells took 7 hours on average (Figure 1E). We speculated that the difference in the timing of mitotic entry reflected a cell cycle-dependence of checkpoint slippage. As a result, some cells in late-S and G2 phases slipped into mitosis after cisplatin exposure, whereas cells treated in G1 and early S phases were effectively prevented from mitotic entry due to checkpoint arrest or cell death. There are several possible mechanisms underlying this observation. First, induction of DNA damage by cisplatin may be less efficient in late S and G2 cells, or alternatively, the DNA damage checkpoint in late S and G2 is inadequate in preventing mitotic entry. Notably, previous studies indicated that an imperfect G2/M DNA damage checkpoint failed to halt the cell cycle with a subthreshold level of DNA damage [20, 21].

### Mitotic cell death is associated with prolonged mitosis, but the duration of mitosis does not predict the cell fate in the subsequent interphase

**Figure 2:**
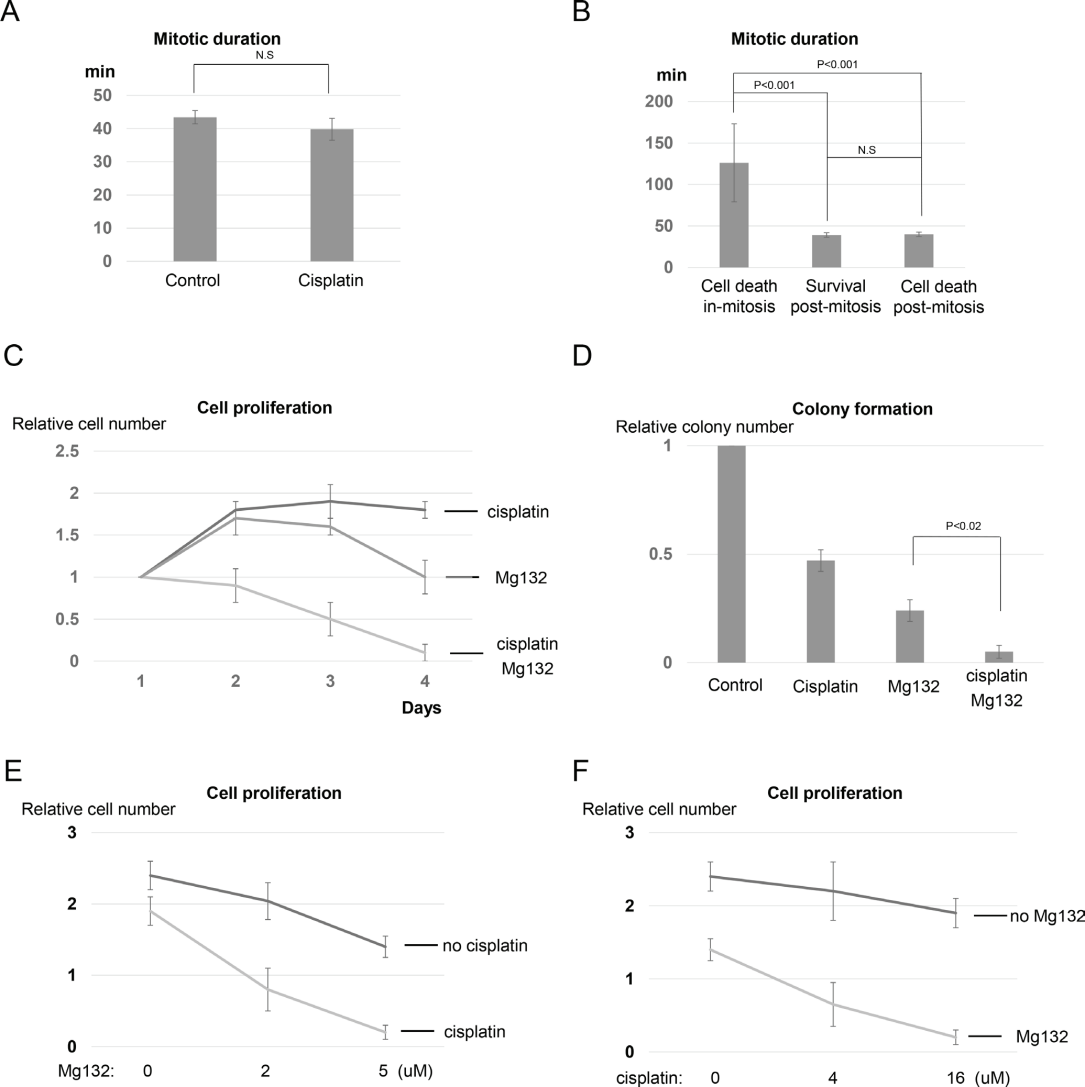
Targeting mitotic exit sensitizes cisplatin response by promoting mitotic cell death (**A**) UM-SCC-38 cells were treated with or without cisplatin as indicated. The average amount of time (in minutes) that UM-SCC-38 cells spent in mitosis is shown. (**B**) The duration of mitosis in three different behavioral groups of UM-SCC-38 cells is shown. (**C**) UM-SCC-38 cells were treated with cisplatin (16 μM) only, Mg132 (5 μM) only, or cisplatin in combination with Mg132 over a period of 4 days. Cell number in each group was measured as described in Materials and Methods. The relative cell number (actual cell number/the starting cell number in day 1) is shown. (**D**) Clonogenic assay was performed as described in Materials and Methods. UM-SCC-38 cells were untreated (control), treated with cisplatin only, Mg132 only, or cisplatin combined with Mg132. (**E**) UM-SCC-38 cells were treated with Mg132 at the indicated concentrations, with or without cisplatin (16 μM). On the fourth day after the treatment, cell numbers were measured as described in Materials and Methods. The relative cell number (actual cell number/the starting cell number in day 1) is shown. (**F**) UM-SCC-38 cells were treated with cisplatin at the indicated concentrations, with or without Mg132 (5 μM). On the fourth day after the treatment, cell numbers were measured as described in Materials and Methods. The relative cell number (actual cell number/the starting cell number in day 1) is shown. In all panels, the mean values and standard errors were calculated from multiple independent experiments, as described in Materials and Methods. *P*-value > 0.05 is considered non-significant (N.S).

Mitotic arrest can result from erratic progression of mitosis and activation of the mitotic spindle checkpoint. Notably, no mitotic arrest was induced by cisplatin treatment, as cells in the control and cisplatin-treated groups spent similar amount of time in mitosis (Figure 2A). We further separated the cisplatin-treated and mitotic-entering cells into three groups based on their subsequent cell fates: died in mitosis, exited mitosis and survived, or exited mitosis and died in the following interphase (Figure 2B). We observed no correlation between mitotic duration and the subsequent cell fate after mitotic exit (Figure 2B). Therefore, mitotic duration does not predict cell death or survival in the subsequent interphase. However, dramatically prolonged mitosis was associated with mitotic death, as cells that destined to die in mitosis spent an average of 126 minutes in mitosis before undergoing cell death (Figure 2B). This finding suggested that delaying mitotic exit may enhance the effectiveness of cisplatin by inducing cell death in mitosis. To directly test this hypothesis, we co-treated UM-SCC-38 cells with Mg132, a proteasome inhibitor known to suppress M-phase exit [22]. The combination of cisplatin and Mg132 resulted in mitotic cell death in 96% of cells, compared to less than 4% with cisplatin alone (Figure S3). Consistently, the cisplatin and Mg132 combination exhibited strong toxicity in UM-SCC-38 cells, as judged by suppression of cell growth and colony formation (Figure 2C and 2D). Furthermore, we utilized multiple doses of cisplatin and Mg132 to further validate the synergy between cisplatin and Mg132, as shown in Figure 2E and 2F for the dose responses.

### Cells exposed to cisplatin during mitosis are hypersensitive

**Figure 3:**
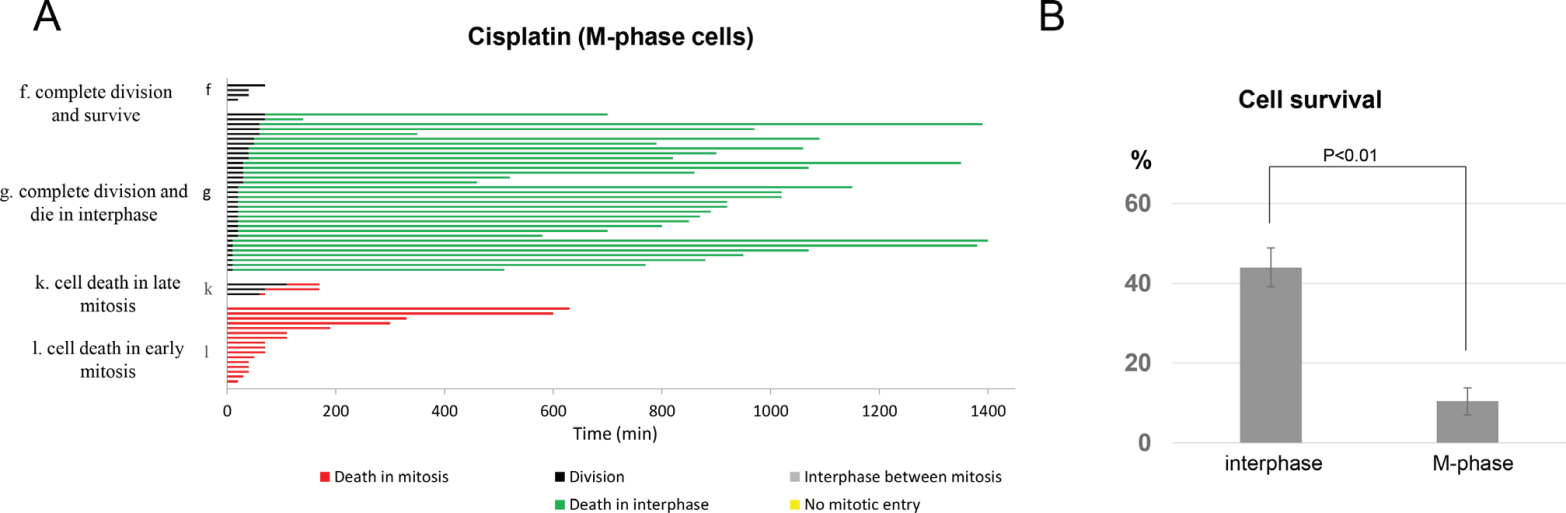
Mitotic cells are hypersensitive to cisplatin (**A**) In the asynchronized UM-SCC-38 population, there were approximately 2–3% cells in mitosis which were identified morphologically under live cell microscopy, and their individual cell fate was collected and analyzed. Each horizontal line represents one cell, with the length of the line corresponding to the duration of a given behavior. The color of the line represents a specific cell behavior as indicated. The y-axis is organized to reflect various cell fates: f. complete division and survive; g. complete division and die in interphase; k. cell death in late mitosis; l. cell death in early mitosis. (**B**) The percentages of cell survival are shown in interphase or M-phase UM-SCC-38 cells treated with cisplatin. In all panels, the mean values and standard errors were calculated from multiple independent experiments, as described in Materials and Methods. *P-*value > 0.05 is considered non-significant (N.S).

It is well known that DNA crosslinks induced by cisplatin interfere with DNA replication and transcription, and thereby, lead to cell death [5, 6]. This widely held view prompted us to examine the fate of cells exposed to cisplatin during mitosis, the cell cycle stage in which DNA replication and transcription are suppressed. Moreover, recent studies revealed that mitotic DNA damage response differs from that of interphase cells, and is often diminished [23, 24]. As collected in Figure 3A, we found that, similar to interphase cells, M-phase cells exhibited multiple fates following cisplatin exposure. However, M-phase cells were extremely sensitive to cisplatin, and the chance of cell survival was markedly reduced in cells exposed to cisplatin in mitosis: 7% survival in M-phase compared to 44% in interphase (Figure 3B). Of the 93% of M-phase cells died after cisplatin treatment, 34% died during mitosis and the other 59% completed cell division, and then died in the subsequent interphase (Figure 3A). Furthermore, 29% of cells died in early mitosis prior to any sign of cell division, whereas 5% of cells died in late stages of mitosis. In light of these results, we concluded that 1) cisplatin exerted cytotoxicity in mitotic cells, presumably independent of DNA replication and transcription, and 2) cisplatin induced cell death in mitotic cells with a much higher potency compared to that in interphase cells.

### Chemoresistant cells are protected from cell death by both checkpoint arrest and slippage

**Figure 4:**
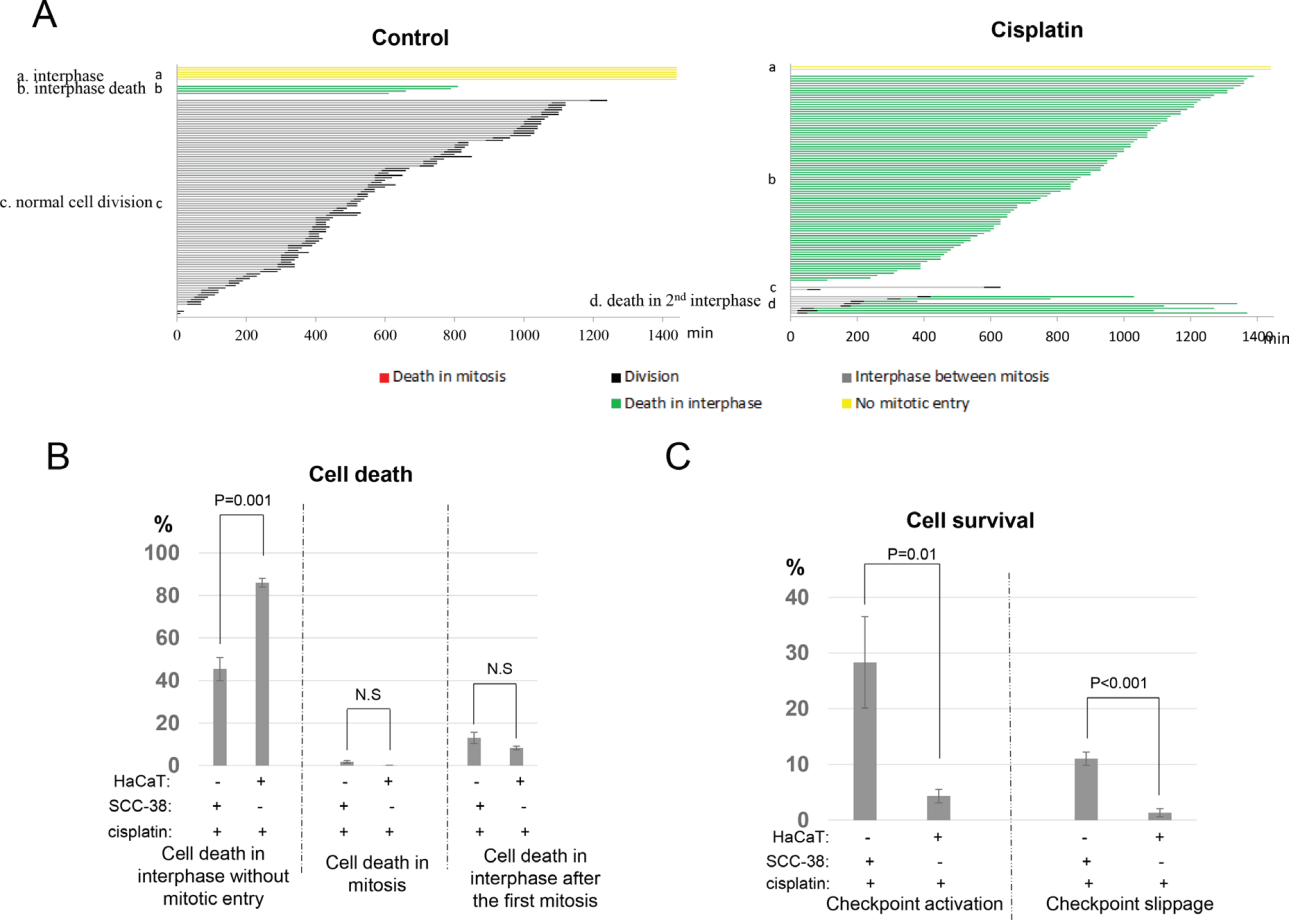
UM-SCC-38 cells are protected by both checkpoint arrest and checkpoint slippage (**A**) Cell fate profiles of HaCaT cells treated with or without cisplatin were quantified. A representative experiment is shown. Each line represents a single cell, and the y-axis is organized to reflect cell fates: a. interphase; b. interphase death; c. normal cell division; d. death in 2nd interphase. (**B**) The induction of cell death by cisplatin in UM-SCC-38 and HaCaT cells. The percentages of cells underwent interphase cell death without mitotic entry, death in mitosis, or death in the subsequent interphase following the first mitosis were compared between these two cell lines. (**C**) The percentages of HaCaT and UM-SCC-38 cells that survived cisplatin treatment by checkpoint activation and checkpoint slippage are shown. In all panels, the mean values and standard errors were calculated from multiple independent experiments, as described in Materials and Methods. *P*-value > 0.05 is considered non-significant (N.S).

To better understand the cisplatin resistance of UM-SCC-38 cells, we comparatively analyzed the cell fate profile of HaCaT, a spontaneously transformed keratinocyte cell line known to be cisplatin sensitive [10]. Interestingly, marked difference in cell fate profiles was noticed between cisplatin-treated UM-SCC-38 and HaCaT cells. As expected, cisplatin induced cell death more efficiently in HaCaT cells (Figure 4A). Approximately 88% of HaCaT cells died in interphase compared to 45% of UM-SCC-38 cells (Figure 4B). By comparison, cell death in mitosis or cell death in interphase after the first mitosis was not increased, but rather moderately reduced in HaCaT cells (Figure 4B). Importantly, and in sharp contrast to UM-SCC-38 cells, much smaller portions of HaCaT cells exhibited checkpoint activation or checkpoint slippage in response to cisplatin (Figure 4C). Only 4% HaCaT cells remained arrested in interphase in comparison to 28% in SCC-38; and 1% of HaCaT cells underwent continued cell cycle progression in the presence of cisplatin, compared to 11% of UM-SCC-38 cells. This comparative study of cell fate profiles highlights the critical role of both checkpoint activation and checkpoint slippage in protecting cells from cell death, which may subsequently lead to cancer resistance.

### Caffeine sensitizes cell death by abolishing both checkpoint activation and checkpoint slippage

**Figure 5:**
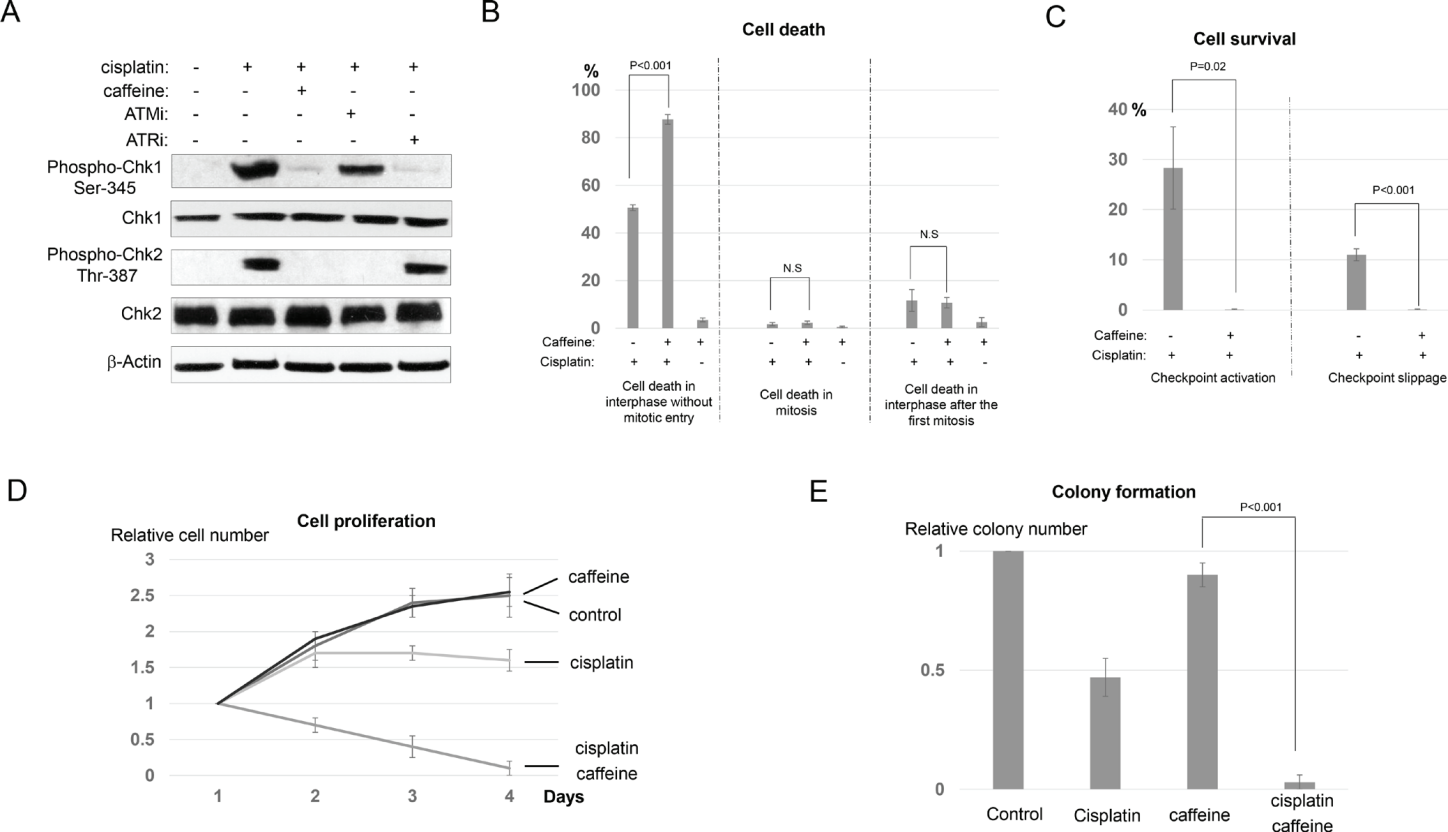
Caffeine sensitizes cell death in conjunction with cisplatin (**A**) UM-SCC-38 cells were treated with cisplatin, caffeine, and specific inhibitors of ATM and ATR (ATMi and ATRi) as described in Materials and Methods. Phosphorylation of Chk1 and Chk2, total Chk1 and Chk2, and β-Actin are shown by immunoblotting. (**B**) UM-SCC-38 cells were treated with cisplatin and caffeine as indicated. The percentages of UM-SCC-38 cells underwent interphase cell death without mitotic entry, death in mitosis, or death in the subsequent interphase following the first mitosis are shown. (**C**) UM-SCC-38 cells were treated with cisplatin and caffeine as indicated. The percentages of UM-SCC-38 cells that survived the treatment by checkpoint activation and checkpoint slippage are shown. (**D**) UM-SCC-38 cells were untreated (control), treated with cisplatin only, caffeine only, or cisplatin in combination with caffeine over a period of 4 days. Cell number in each group was measured as described in Materials and Methods. The relative cell number (actual cell number/the starting cell number in day 1) is shown. (**E**) Clonogenic assay was performed as described in Materials and Methods. UM-SCC-38 cells were untreated (control), treated with cisplatin only, caffeine only, or cisplatin combined with caffeine. In all panels, the mean values and standard errors were calculated from multiple independent experiments, as described in Materials and Methods. *P*-value > 0.05 is considered non-significant (N.S).

It has been suggested that targeting the cellular DDR pathway provides a valid opportunity to reduce the resistance of cancer cells to radiation and chemotherapy. In particular, it is typically thought that disruption of the DNA damage checkpoint will allow cell cycle progression after DNA damage. And subsequently, cell division with unrepaired DNA damage leads to further accumulation of DNA damage, mitotic defects, and eventually, cell death. We sought to reveal how checkpoint disruption would affect the determination of cell fate choices in UM-SCC-38 cells treated with cisplatin. We first confirmed that caffeine, a well characterized inhibitor of ATM and ATR, effectively silenced DNA damage checkpoint signaling induced by cisplatin in UM-SCC-38 cells, as evidenced by decreased phosphorylation of Chk1 and Chk2 (Figure 5A). Interestingly, caffeine treatment resulted in significantly increased cell death in interphase, but not in mitosis or the second interphase following the first mitosis (Figure 5B and S4). This effect of caffeine strongly supported that checkpoint disruption directly sensitizes cell death without either mitotic entry or accumulation of DNA damage due to mitotic defects. As a control, caffeine alone did not induce cell death in UM-SCC-38 cells (Figure S5A–S5C, 5B). We then compared the portions of surviving cells between the groups of cisplatin alone and cisplatin/caffeine combination. As expected, caffeine abolished the portion of interphase arrested cells (Figure 5C), presumably by suppressing the ATM/ATR-mediated DNA damage checkpoint. To our surprise, caffeine treatment also completely eliminated the portion of checkpoint slippage, so essentially no cell was able to successfully complete cell division in the presence of caffeine and cisplatin (Figure 5C). Collectively, caffeine treated UM-SCC-38 cells responded to cisplatin in a manner similar to the chemosensitive HaCaT cells. Finally, we confirmed that caffeine greatly enhanced the efficacy of cisplatin in UM-SCC-38 cells using both cell proliferation and clonogenic assays (Figure 5D and 5E).

### Inhibition of ATR, but not ATM, sensitizes interphase cell death

Caffeine inhibits both ATM and ATR, two upstream DDR kinases. It has been well illustrated that ATM and ATR, though sharing great similarity in structural elements and substrate recognition, respond to different types of DNA lesions and are activated by distinct mechanisms [8]. To better clarify the potential involvement of ATM and ATR in cisplatin response, we utilized specific inhibitors that selectively target either ATM or ATR. As confirmed in Figure 5A, Ku55933 (ATMi) inhibited phosphorylation of Chk2 in response to cisplatin, whereas VE-821 (ATRi) disrupted ATR-dependent phosphorylation of Chk1.

**Figure 6:**
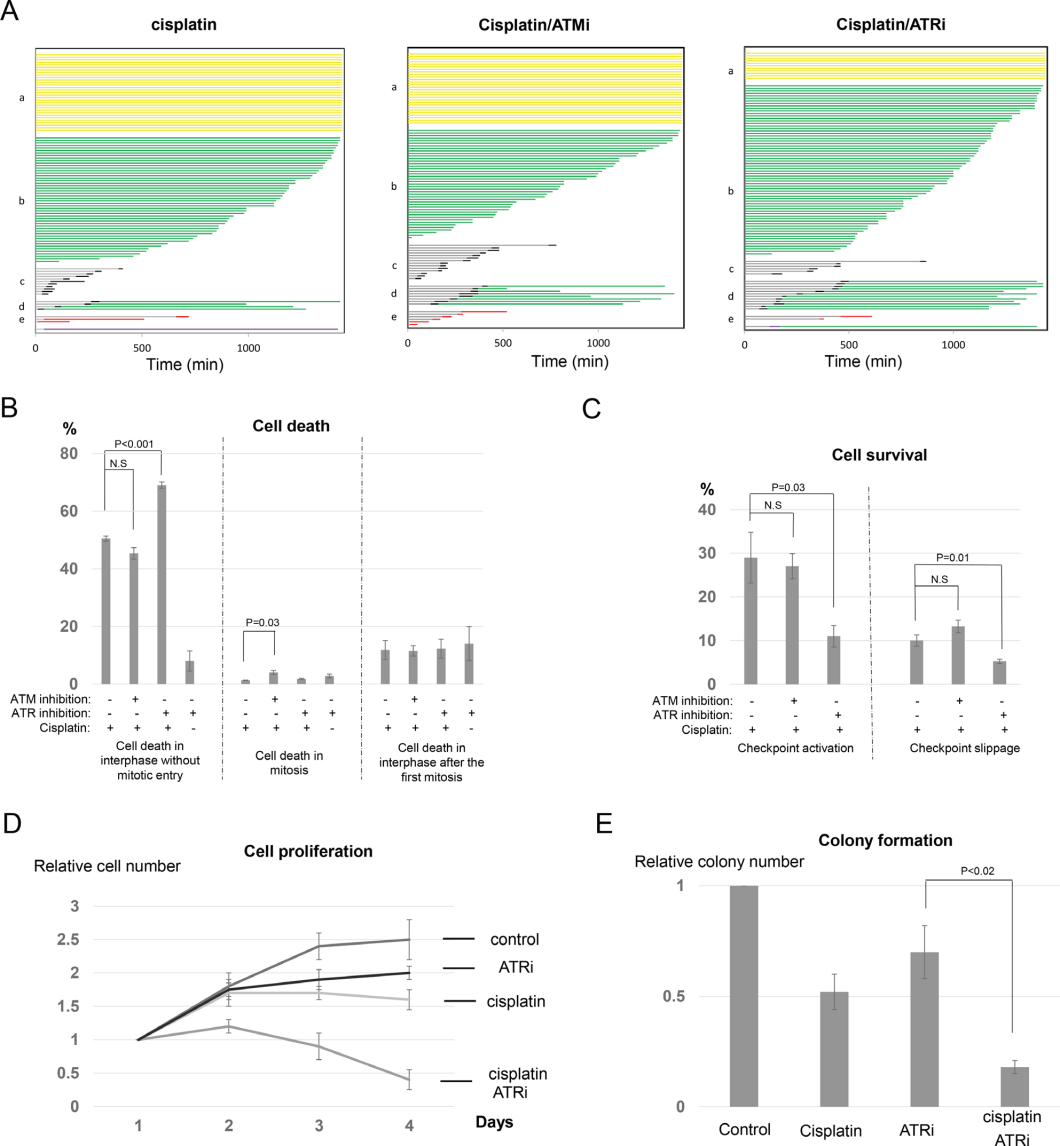
Inhibition of ATR, but not ATM, sensitizes cisplatin treatment (**A**) UM-SCC-38 cells were treated with cisplatin and ATM/ATR inhibitors as indicated. Cell fate profiles were quantified, and a representative experiment is shown. Each horizontal line represents one cell, with the length of the line corresponding to the duration of a given behavior. The color of the line represents a specific cell behavior as indicated. The y-axis is organized to reflect various cell fates: a. interphase; b. interphase death; c. normal cell division; d. death in 2nd interphase; e. mitotic cell death. (**B**) UM-SCC-38 cells were treated with cisplatin and ATM/ATR inhibitors as indicated. The percentages of UM-SCC-38 cells underwent interphase cell death without mitotic entry, death in mitosis, or death in the subsequent interphase following the first mitosis are shown. (**C**) UM-SCC-38 cells were treated with cisplatin and ATM/ATR inhibitors as indicated. The percentages of UM-SCC-38 cells that survived the treatment via checkpoint activation and checkpoint slippage are shown. (**D**) UM- SCC-38 cells were untreated (control), treated with cisplatin only, ATR inhibitor only, or cisplatin and ATR inhibitor over a period of 4 days. Cell number in each group was measured as described in Materials and Methods. The relative cell number (actual cell number/the starting cell number in day 1) is shown. (**E**) Clonogenic assay was performed as described in Materials and Methods. UM-SCC-38 cells were untreated (control), treated with cisplatin only, ATR inhibitor only, or cisplatin combined with ATR inhibitor. In all panels, the mean values and standard errors were calculated from multiple independent experiments, as described in Materials and Methods. *P-*value > 0.05 is considered non-significant (N.S).

To our surprise, ATM inhibition did not significantly alter the profile of cell fate choices after cisplatin treatment (Figure 6A). We observed little difference in neither the induction of interphase cell death nor the portions of surviving cells via checkpoint activation (interphase arrest) or checkpoint slippage (Figure 6B and 6C). A minor induction of mitotic cell death was detected with ATM inhibition (Figure 6B). Unlike ATM inhibition, ATR inhibition in conjunction with cisplatin resulted in interphase cell death in approximately 70% of cells, compared to 50% in the cisplatin only group. Moreover, ATR inhibition substantially reduced the number of cells that were arrested in interphase or underwent checkpoint slippage (Figure 6C). As a control, this ATR inhibitor alone exhibited a moderate effect on the induction of cell death (Figure 6B and S6). The impact of ATR inhibition on the cisplatin treated cells resembled that of caffeine, suggesting that ATR, rather than ATM, plays a major role in cell fate determination after cisplatin treatment. Inspired by this conclusion, we further confirmed that ATR inhibition synergistically sensitized UM-SCC-38 cells to cisplatin in cell proliferation and clonogenic assays (Figure 6D and 6E). Thus, ATR-mediated checkpoint pathway presents a promising target to improve the therapeutic outcome of cisplatin.

## DISCUSSION

Quantitative measurement of individual cell fate with live-cell imaging can reveal detailed information with respect to how cell fate choices are determined. In turn, the knowledge about cell fate choices will help us understand cancer resistance and improve treatment efficacy. In this study we profiled the outcome of cisplatin treatment in chemoresistant UM-SCC-38 cells. A significantly smaller portion of UM-SCC-38 cells died after the treatment when compared to HaCaT, a non-tumorigenic keratinocyte cell line. Interestingly, in both UM-SCC-38 and HaCaT lines, the majority of cell death occurred in interphase without mitotic entry. By comparison, only small portions of cells either entered and then died in mitosis, or completed cell division and then died in the subsequent interphase. Analysis of the surviving UM-SCC-38 cells revealed the co-existence of checkpoint arrest and checkpoint slippage: some surviving cells remained arrested in interphase without mitotic entry, while another group of cells underwent active cell division without detectable delay in either interphase or M-phase. Hence, our study revealed a complex pattern of cell fate choices in cancer cells treated with cisplatin. A similar pattern of cell fate determination was also observed in another cisplatin-resistant head and neck cancer cell line UM-SCC-11B (Figure S7).

We examined the length of mitosis in cells that entered mitosis after cisplatin treatment. Surprisingly, cisplatin did not cause prolonged mitosis in cells treated prior to mitotic entry, indicating the absence of vital mitotic defects or activation of the mitotic spindle checkpoint. In principle, cells that entered mitosis after therapeutic treatment may die in mitosis, die after mitotic exit, or complete mitosis and survive. As reported in this study, all these choices existed in cisplatin-treated UM-SCC-38 cells. We then asked if the duration of mitosis predicted cell death or survival in the subsequent interphase. However, no significant difference was observed in the length of mitosis regardless of the cell fate after mitotic exit, as groups of post-mitotic cell death and survival spent 40 and 39 minutes, respectively, in the mitotic phase. Very interestingly, we found that the small portion of cisplatin-treated cells that entered, and then died in mitosis typically spent more than 2 hours in mitosis prior to cell death. Inspired by this association between the prolonged mitotic progression and mitotic cell death, we showed a surprisingly strong synergy between cisplatin and Mg132, a proteasome inhibitor known to suppress mitotic exit. As expected, when co-treated with cisplatin and Mg132, the vast majority of cells were trapped in mitosis and underwent mitotic cell death. A rather surprising implication of this result is that, while approximately 25% cells stay arrested (and alive) when treated with cisplatin alone, this portion of cells were apparently “forced” into mitosis and subsequently underwent cell death when treated with both cisplatin and Mg132. Thus, our study suggested a promising strategy of combinatorial therapy using cisplatin and Mg132, which shall be further evaluated in experimental or clinical studies. Consistently, previous studies also suggested the therapeutic potential of Mg132 by either directly inducing cell death, or reversing the resistance of cancer cells to other drugs, including cisplatin [25–28].

The pattern of cell fate choices differed remarkably in cells exposed to cisplatin during mitosis. Collectively, mitotic cells were more sensitive to cisplatin, and the majority of these cells died in mitosis or after mitotic exit. Thus, our finding adds to the existing knowledge of how cisplatin exerts its toxicity in the cell: in addition to blocking DNA replication and transcription, cisplatin may also induce DNA damage in mitotic cells and interfere with mitotic progression. Moreover, recent studies showed that the molecular pathways of DNA repair and DNA damage checkpoint are largely silenced during mitosis [23, 24]. It has been also suggested that the mitotic suppression of DNA repair is beneficial as mitotic DNA repair may lead to chromosomal instability, e.g., via telomere fusion [29]. Therefore, the hypersensitivity to DNA damage is a desirable choice for mitotic cells that lack the capability of DNA repair.

As the cellular DDR plays a key role in cell fate determination after DNA damage, it has been proposed that targeting the DDR may offer a powerful tool to overcome chemoresistance. In support of this notion, we found that UM-SCC-38 cells treated with caffeine, an inhibitor of ATM and ATR, exhibited greatly enhanced cell death after cisplatin treatment. Contrary to the common assumption that checkpoint disruption would lead to cell death by allowing mitotic entry with DNA damage, our study showed that the caffeine and cisplatin combination almost exclusively induced cell death in interphase without mitotic entry. As expected, caffeine suppressed checkpoint activation after cisplatin treatment, and abolished the portion of cell survival via interphase arrest. Moreover, and perhaps counterintuitively, caffeine treatment also eliminated the portion of checkpoint slippage. We speculate that caffeine may prevent checkpoint slippage at least partially by suppressing DNA repair, as supported by several recent studies [30–32].

As caffeine simultaneously inhibits ATM and ATR, we further advanced the study using inhibitors that specifically target either one of these kinases. Similar to caffeine, ATR inhibition reduced cell survival by preventing checkpoint arrest and checkpoint slippage, and enhancing cell death in interphase. By comparison, ATM inhibition exhibited no significant effect on cell death or survival. Therefore, the effect of caffeine in sensitizing the cisplatin treatment is largely conferred through ATR inhibition. This finding is interesting given that both ATM and ATR have been linked to the cisplatin response, and that pharmacological inhibition of both has been implicated in anti-cancer treatment [5, 12–14]. It is well-established that ATR regulates DNA replication, cell cycle checkpoints and DNA repair [33, 34]. Future efforts are required to delineate the molecular detail underlying the role of ATR in cisplatin resistance. Moreover, it should be noted that the effect of ATR inhibition appeared less profound compared to that of caffeine, which possibly implies additional targets of caffeine, as suggested previously [35].

In summary, we reported here the first quantitative analysis of cell fate determination in cancer cells treated with cisplatin. The results revealed new insights into chemoresistance and the potential of combination therapy using cisplatin and agents that block mitotic exit or the DNA damage checkpoint. Our study focused on the initial response to cisplatin, and a long-term examination into the subsequent cycles of cell proliferation shall be carried out in future studies.

## MATERIALS AND METHODS

### Cell culture and drug treatment

As in our previous study [10], UM-SCC-38 cells were grown in Dulbecco's modified Eagle's medium (DMEM, Sigma Chemical Co., St. Louis, MO.) supplemented with 10% fetal bovine serum (Hyclone Laboratories, Logan, UT), and HaCaT cells were passaged using DMEM medium lacking calcium (Invitrogen, Grand Island, NY.) supplemented with 10% fetal bovine serum. These cell lines were previously characterized genetically and morphologically (10,11). Cisplatin (cis-diammineplatinum (II) dichloride) was purchased from Sigma (St. Louis, MO) and used at a final concentration of 16 μM unless specified. ATM/ATR inhibitors used in this study include caffeine (Sigma, St. Louis, MO), KU55933 (EMD Chemicals), and VE-821 (SELLECK Chemical LLC). The final concentrations of these inhibitors in cell culture are 4 mM for caffeine, 20 μM for KU55933, and 10 μM for VE-821. Mg132 was purchased from Sigma (St. Louis, MO) and used at a final concentration of 5 μM unless specified.

### Cell proliferation and clonogenic assays

As in our previous studies [10, 36], cells with or without drug treatment were incubated for 1–4 days. The numbers of viable cells were counted using a hemocytometer. For clonogenic assays, cells were seeded into 6-well plates at a density of 1,000 cells per well. After 24 hours, cells were treated with or without drugs. After incubation for 2 weeks, cells were then fixed in 1% glutaraldehyde for 30 minutes, stained with 5% crystal violet, and counted for colony numbers.

### Immunoblotting

As described previously [37], samples were denatured by boiling in 2X Laemmli sample buffer, resolved by sodium dodecyl sulfate-polyacrylamide gel electrophoresis (SDS-PAGE), and then electrotransfered to Polyvinylidene Difluoride (PVDF) membranes (Millipore, Billerica, MA). Membranes were incubated in blocking buffer (10 mM Tris HCl pH 7.5, 150 mM NaCl, 0.05% Tween 20, and 5% non-fat milk) for 1 hour, and then with primary antibodies for 2 hours. Phospho-Chk1, and Chk2 antibodies were purchased from Cell Signaling Technology (Danvers, MA), and Chk1, phospho-Chk2, and β-actin antibodies were purchased from Abcam (Cambridge, MA). The membrane was then incubated with horseradish peroxidase (HRP)-conjugated secondary antibodies (Sigma-Aldrich, Louis, MO) for 1 hr, and then detected using an Enhanced Chemiluminescence (ECL) substrate kit (Pierce).

### Live cell imaging and data analysis

Two days prior to microscopy cells were passaged and seeded in a 6-well plate (Celltreat, China), at roughly 50 to 80% confluence. Live cell imaging was performed using the Marianas Live Cell system based around a Zeiss Axiovert 200M microscope stand, and the SlideBook6 software (Intelligent Imaging Innovations, Inc, Denver, CO.). Images were collected every 10 minutes for 24 hours with 10X objective lens magnification. Once the live cell microscopy was completed, the captured images were loaded into SlideBook Reader Software (Intelligent Imaging Innovations). Under each condition, one hundred cells were manually tracked for cell fates in the experiment. Cell behaviors were entered into Microsoft Excel Spreadsheet to generate cell profile graphs, as illustrated in a previous study [38] (Figures S1 and S2). Statistical significance was analyzed using an unpaired 2-tailed Student's *t*-test. The values are presented as the means ± standard errors. A *p*-value < 0.05 was considered statistically significant.

## SUPPLEMENTARY MATERIALS FIGURES



## References

[1] Lord CJ, Ashworth A (2012). The DNA damage response and cancer therapy. Nature.

[2] Jackson SP, Bartek J (2009). The DNA-damage response in human biology and disease. Nature.

[3] Bolderson E, Richard DJ, Zhou BBS, Khanna KK (2009). Recent Advances in Cancer Therapy Targeting Proteins Involved in DNA Double-Strand Break Repair. Clin Cancer Res.

[4] Al-Ejeh F, Kumar R, Wiegmans A, Lakhani SR, Brown MP, Khanna KK (2010). Harnessing the complexity of DNA-damage response pathways to improve cancer treatment outcomes. Oncogene.

[5] Galluzzi L, Senovilla L, Vitale I, Michels J, Martins I, Kepp O, Castedo M, Kroemer G (2012). Molecular mechanisms of cisplatin resistance. Oncogene.

[6] Caponigro F, Milano A, Basile M, Ionna F, Iaffaioli RV (2006). Recent advances in head and neck cancer therapy: the role of new cytotoxic and molecular-targeted agents. Curr Opin Oncol.

[7] Zhou BBS, Elledge SJ (2000). The DNA damage response: putting checkpoints in perspective. Nature.

[8] Shiloh Y (2003). ATM and related protein kinases: Safeguarding genome integrity. Nature Reviews Cancer.

[9] Sancar A, Lindsey-Boltz LA, Unsal-Kacmaz K, Linn S (2004). Molecular mechanisms of mammalian DNA repair and the DNA damage checkpoints. Annu Rev Biochem.

[10] Wang L, Mosel AJ, Oakley GG, Peng A (2012). Deficient DNA damage signaling leads to chemoresistance to Cisplatin in oral cancer. Mol Cancer Ther.

[11] Brenner JC, Graham MP, Kumar B, Saunders LM, Kupfer R, Lyons RH, Bradford CR, Carey TE (2010). Genotyping of 73 UM-SCC head and neck squamous cell carcinoma cell lines. Head Neck.

[12] Prendergast AM, Cruet-Hennequart S, Shaw G, Barry FP, Carty MP (2011). Activation of DNA damage response pathways in human mesenchymal stem cells exposed to cisplatin or gamma-irradiation. Cell Cycle.

[13] Zhao H, Piwnica-Worms H (2001). ATR-mediated checkpoint pathways regulate phosphorylation and activation of human Chk1. Mol Cell Biol.

[14] Pabla N, Huang S, Mi QS, Daniel R, Dong Z (2008). ATR-Chk2 signaling in p53 activation and DNA damage response during cisplatin-induced apoptosis. J Biol Chem.

[15] Shaltiel IA, Krenning L, Bruinsma W, Medema RH (2015). The same, only different - DNA damage checkpoints and their reversal throughout the cell cycle. J Cell Sci.

[16] Bartek J, Lukas J (2007). DNA damage checkpoints: from initiation to recovery or adaptation. Curr Opin Cell Biol.

[17] Clemenson C, Marsolier-Kergoat MC (2009). DNA damage checkpoint inactivation: Adaptation and recovery. DNA Repair.

[18] Syljuasen RG (2007). Checkpoint adaptation in human cells. Oncogene.

[19] Peng A (2013). Working hard for recovery: mitotic kinases in the DNA damage checkpoint. Cell Biosci.

[20] Deckbar D, Birraux J, Krempler A, Tchouandong L, Beucher A, Walker S, Stiff T, Jeggo P, Lobrich M (2007). Chromosome breakage after G2 checkpoint release. J Cell Biol.

[21] Krempler A, Deckbar D, Jeggo PA, Lobrich M (2007). An imperfect G(2)/M checkpoint contributes to chromosome instability following irradiation of S and G(2) phase cells. Cell Cycle.

[22] Skoufias DA, Indorato RL, Lacroix F, Panopoulos A, Margolis RL (2007). Mitosis persists in the absence of Cdk1 activity when proteolysis or protein phosphatase activity is suppressed. J Cell Biol.

[23] Heijink AM, Krajewska M, van Vugt MA (2013). The DNA damage response during mitosis. Mutat Res.

[24] Giunta S, Belotserkovskaya R, Jackson SP (2010). DNA damage signaling in response to double-strand breaks during mitosis. J Cell Biol.

[25] Guo N, Peng Z (2013). MG132, a proteasome inhibitor, induces apoptosis in tumor cells. Asia Pac J Clin Oncol.

[26] Zhang Y, Shi Y, Li X, Du R, Luo G, Xia L, Du W, Chen B, Zhai H, Wu K, Fan D (2008). Proteasome inhibitor MG132 reverses multidrug resistance of gastric cancer through enhancing apoptosis and inhibiting P-gp. Cancer Biol Ther.

[27] Ma J, Yu L, Tian J, Mu Y, Lv Z, Zou J, Li J, Wang H, Xu W (2014). MG132 reverse the malignant characteristics of hypopharyngeal cancer. Mol Med Rep.

[28] Dang L, Wen F, Yang Y, Liu D, Wu K, Qi Y, Li X, Zhao J, Zhu D, Zhang C, Zhao S (2014). Proteasome inhibitor MG132 inhibits the proliferation and promotes the cisplatin-induced apoptosis of human esophageal squamous cell carcinoma cells. Int J Mol Med.

[29] Orthwein A, Fradet-Turcotte A, Noordermeer SM, Canny MD, Brun CM, Strecker J, Escribano-Diaz C, Durocher D (2014). Mitosis inhibits DNA double-strand break repair to guard against telomere fusions. Science.

[30] Zelensky AN, Sanchez H, Ristic D, Vidic I, van Rossum-Fikkert SE, Essers J, Wyman C, Kanaar R (2013). Caffeine suppresses homologous recombination through interference with RAD51-mediated joint molecule formation. Nucleic Acids Res.

[31] Tsabar M, Eapen VV, Mason JM, Memisoglu G, Waterman DP, Long MJ, Bishop DK, Haber JE (2015). Caffeine impairs resection during DNA break repair by reducing the levels of nucleases Sae2 and Dna2. Nucleic Acids Res.

[32] Sabisz M, Skladanowski A (2008). Modulation of cellular response to anticancer treatment by caffeine: inhibition of cell cycle checkpoints, DNA repair and more. Curr Pharm Biotechnol.

[33] Flynn RL, Zou L (2011). ATR: a master conductor of cellular responses to DNA replication stress. Trends Biochem Sci.

[34] Nam EA, Cortez D (2011). ATR signalling: more than meeting at the fork. Biochem J.

[35] Cortez D (2003). Caffeine inhibits checkpoint responses without inhibiting the ataxia-telangiectasia-mutated (ATM) and ATM- and Rad3-related (ATR) protein kinases. J Biol Chem.

[36] Wang L, Luong VQ, Giannini PJ, Peng A (2014). Mastl kinase, a promising therapeutic target, promotes cancer recurrence. Oncotarget.

[37] Peng A, Wang L, Fisher LA (2011). Greatwall and Polo-like kinase 1 coordinate to promote checkpoint recovery. J Biol Chem.

[38] Gascoigne KE, Taylor SS (2008). Cancer cells display profound intra- and interline variation following prolonged exposure to antimitotic drugs. Cancer Cell.

