# Supplementary material for: Cell fate determination in cisplatin resistance and chemosensitization
Source: Oncotarget. 2016 Mar 16;7(17):23383–94. doi: 10.18632/oncotarget.8110 (PMC5029634; doi:10.18632/oncotarget.8110)
Supplement: Supplementary file 1 [file oncotarget-07-23383-s001.pdf]

Cell Fate Determination in Cisplatin Resistance and Chemosensitization

Supplementary Materials

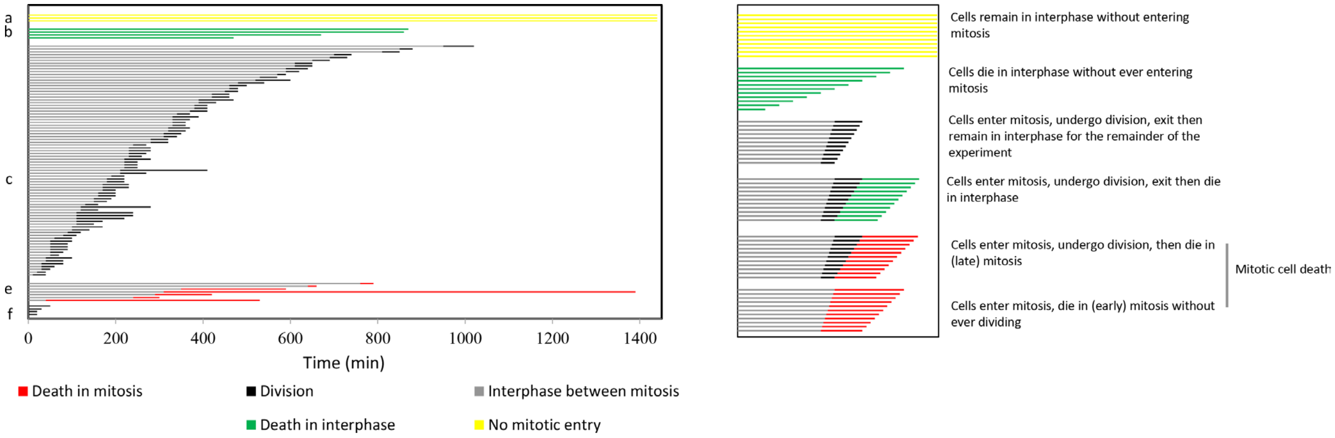

Supplementary Figure S1: Profiling the cell fate choices. Cell fate choices were monitored by live cell imaging, as described in Methods and Materials. This figure illustrates how cell profile was constructed. Each horizontal line represents one cell, with the length of the line corresponding to the duration of a given behavior. The color of the line represents a specific cell behavior as indicated. The y-axis is organized to reflect various cell fates: a. interphase; b. interphase death; c. normal cell division; d. death in 2nd interphase; e. mitotic cell death; f. complete division and survive; g. complete division and die in interphase; k. cell death in late mitosis; l. cell death in early mitosis.

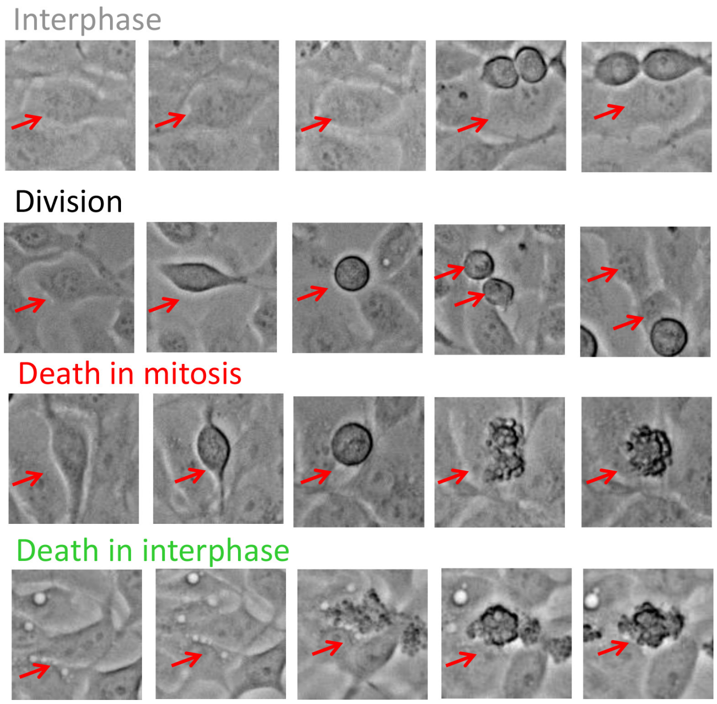

Supplementary Figure S2: Various cell fates post cisplatin treatment. Examples of the behaviors of UM-SCC-38 cells observed using time-lapse microscope are shown.

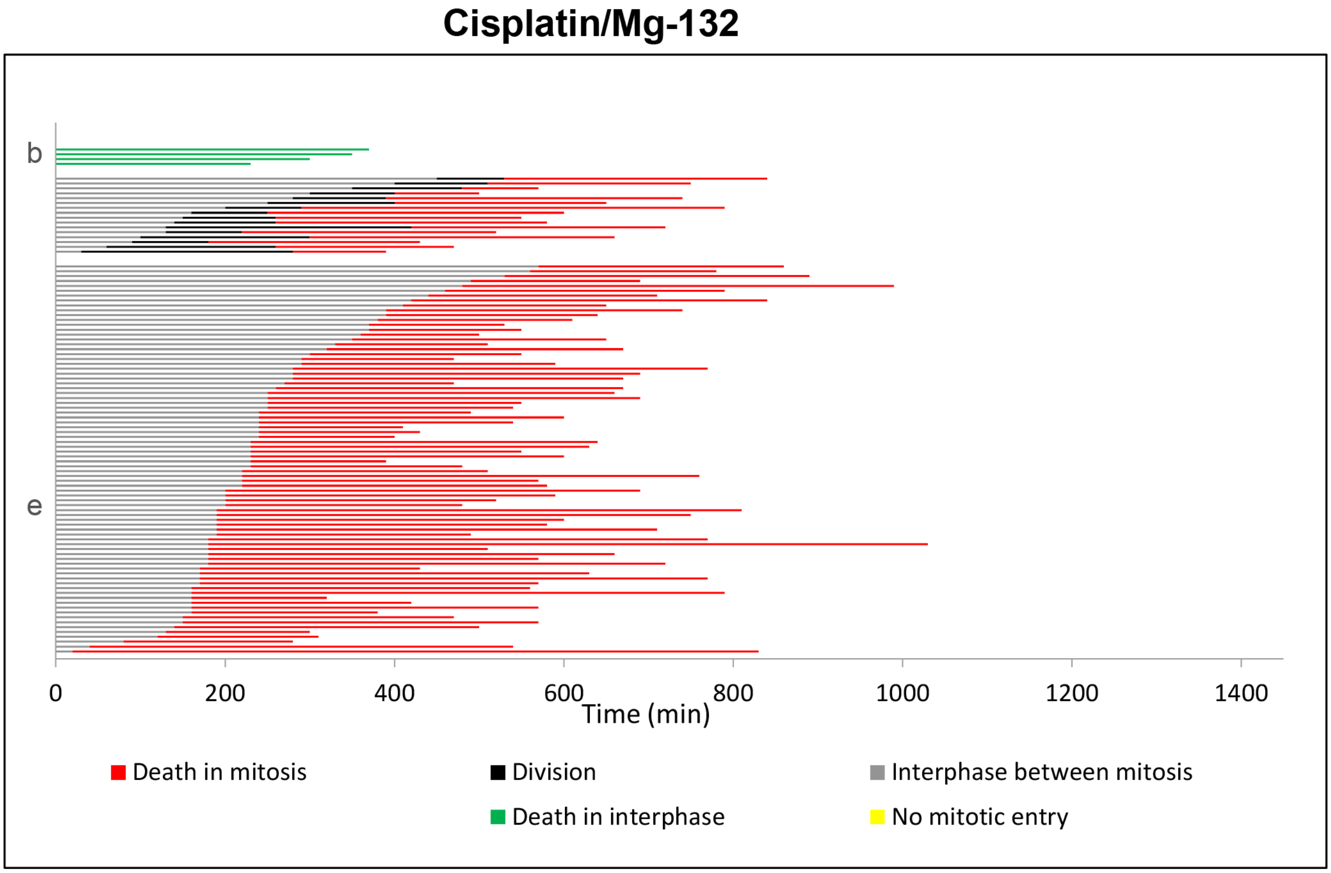

Supplementary Figure S3: Cell fate choices of UM-SCC-38 cells treated with cisplatin and Mg-132. The cell fate profile of UM-SCC-38 cells that were treated with a combination of 16 µM cisplatin and 5 µM Mg-132.

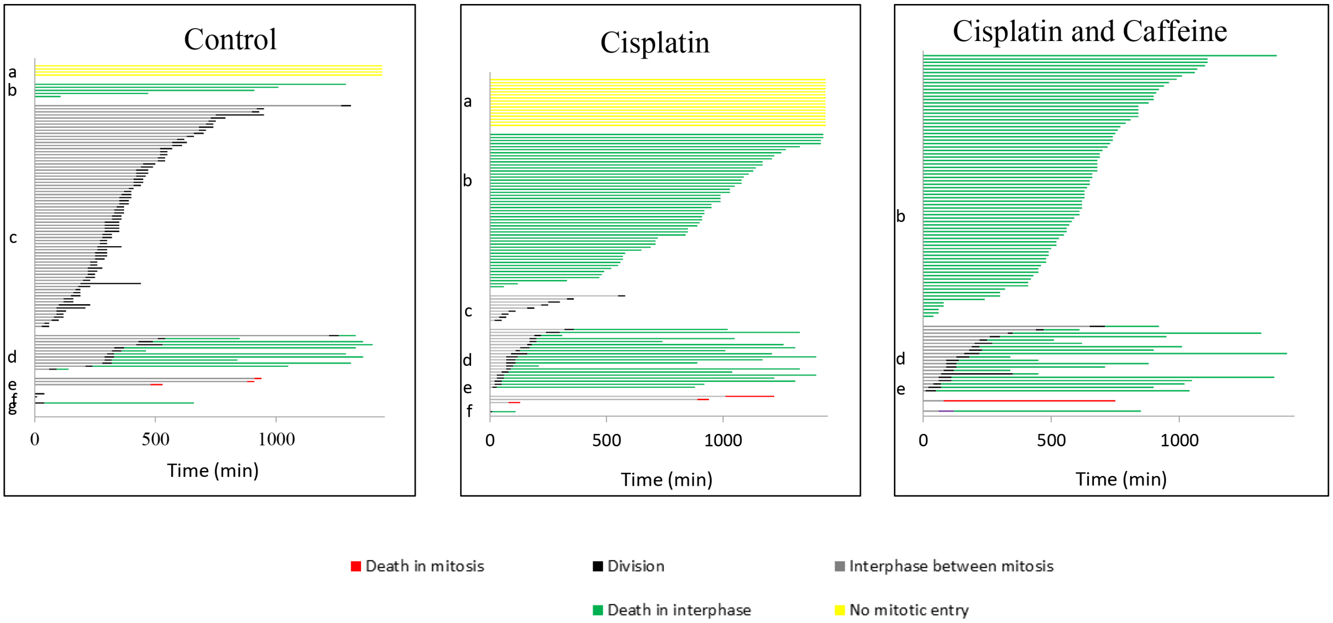

Supplementary Figure S4: Cell fate choices of UM-SCC-38 cells treated with cisplatin and caffeine. Cell fate profiles of UM-SCC-38 cells following exposure to cisplatin only or cisplatin plus caffeine. UM-SCC-38 cells without cisplatin or caffeine treatment were included as control (left graph). Each horizontal line represents one cell, with the length of the line corresponding to the duration of a given behavior. The color of the line represents a specific cell behavior as indicated. The y-axis is organized to reflect various cell fates: a. interphase; b. interphase death; c. normal cell division; d. death in 2nd interphase; e. mitotic cell death; f. complete division and survive; g. complete division and die in interphase.

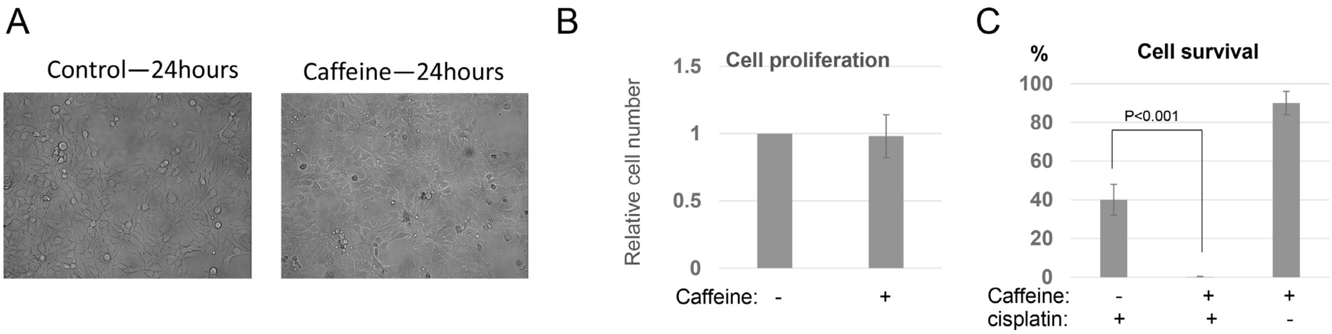

Supplementary Figure S5: Caffeine-treatment alone does not induce cell death in UM-SCC-38 cells. (A) UM-SCC-38 cells were treated with or without caffeine. We observed no significant effect on cell proliferation or death. Representative images of cells with or without caffeine-treatment are shown. (**B**) As in panel A, cell numbers were counted after 24 hr treatment with or without caffeine. (**C**) UM-SCC-38 cells were treated with cisplatin and caffeine as indicated. Cell fates were analyzed as in Figure S4. The percentages of cells that survived the treatment are shown.

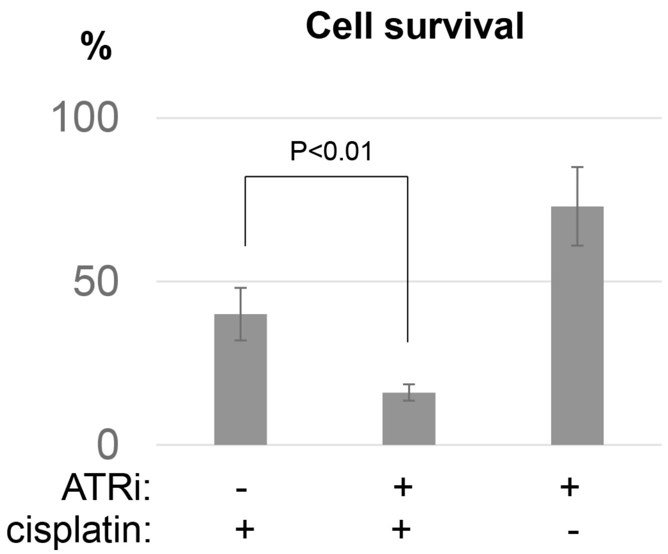

Supplementary Figure S6: UM-SCC-38 cell survival in response to cisplatin and ATRi. UM-SCC-38 cells were treated with cisplatin and ATRi as indicated. Cell fates were analyzed as in Figure 6A. The percentages of cells that survived the treatment are shown.

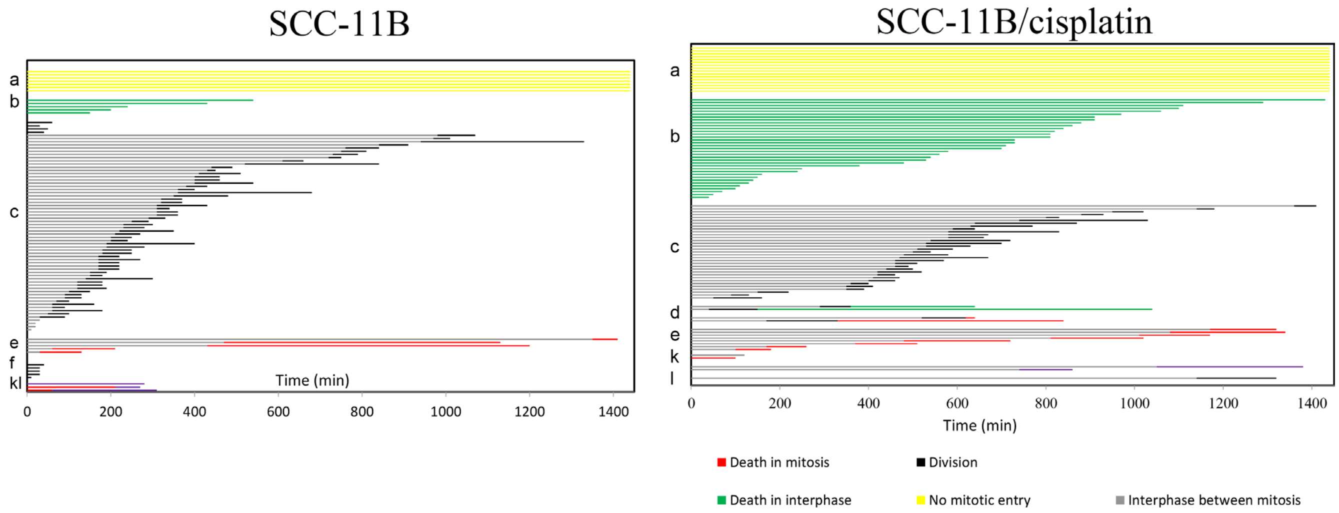

Supplementary Figure S7: Cell fate choices of UM-SCC-11B cells treated with cisplatin. The cell fate profile of UM-SCC-11B cells that were treated with or without cisplatin. Each horizontal line represents one cell, with the length of the line corresponding to the duration of a given behavior. The color of the line represents a specific cell behavior as indicated. The y-axis is organized to reflect various cell fates: a. interphase; b. interphase death; c. normal cell division; d. death in 2nd interphase; e. mitotic cell death; f. complete division and survive; g. complete division and die in interphase; k. cell death in late mitosis; l. cell death in early mitosis.
